# Supplementary material for: Cycle Threshold (Ct) Values of SARS-CoV-2 Detected with the GeneXpert® System and a Mutation Associated with Different Target Gene Failure
Source: Curr Issues Mol Biol. 2023 May 7;45(5):4124–34. doi: 10.3390/cimb45050262 (PMC10217001; doi:10.3390/cimb45050262)
Supplement: Supplementary file 1 [file cimb-45-00262-s001.zip › cimb-2356239-supplementary.pdf]

Table S1. Oligonucleotide primers and probe used for analyzing the SARS-CoV-2 nucleocapsid gene.

Name	Description	Nucleotide No.	Sequence (5' to 3')	Gene site	Expected size (bp)	Reference
N-1F	Forward Primer	21	CTG CTC TTG CTT TGC TGC TGC	28923-28943	436	
N-1R	Reverse Primer	21	GTT TTG TAT GCG TCA ATA TGC	29358-29338		
NIID-N-F2	Forward Primer	20	AAA TTT TGG GGA CCA GGA AC	29125-29144	158	NIID_2019-nCOV_N_F2
NIID-N-R2	Reverse Primer	20	TGG CAG CTG TGT AGG TCA AC	29282-29263		NIID_2019-nCOV_N_R2
CDC-N2-F	Forward Primer	20	TTA CAA ACA TTG GCC GCA AA	29164-29183	67	CDC 2019-nCoV_N2-F
CDC-N2-R	Reverse Primer	18	GCG CGA CAT TCC GAA GAA	29230-29213		CDC 2019-nCoV_N2-R
CDC-N2-P	Probe	23	FAM-ACA ATT TGC CCC CAG CGC TTC AG-BHQ1	29188-29210		CDC 2019-nCoV_N2-P

NIID: National Institute of Infectious Diseases
CDC: Centers for Disease Control and Prevention
F: Forward primer
R: Reverse primer
P: Probe

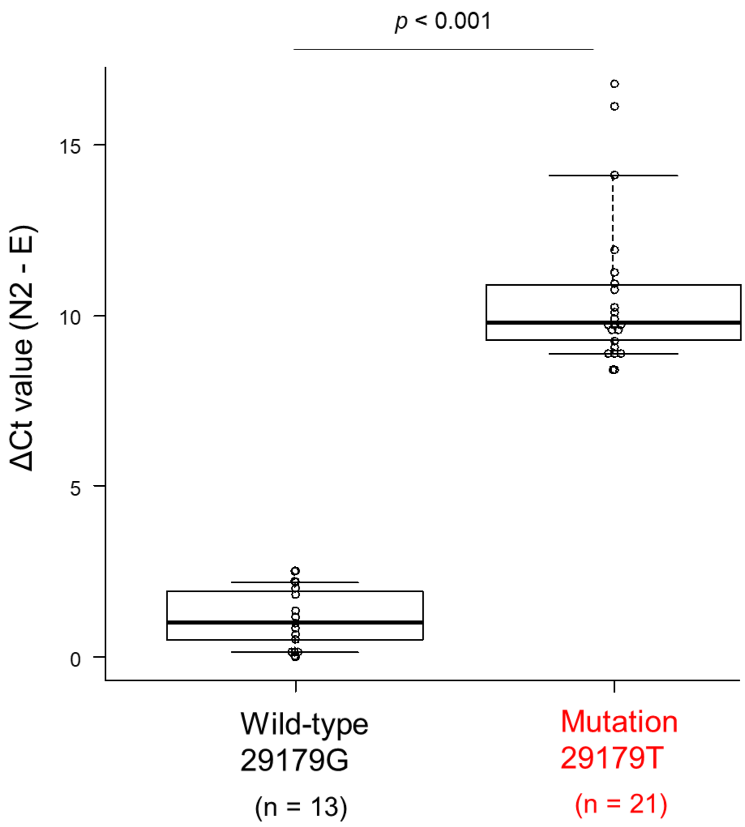

Figure S1. Comparison of the Ct delay calculated as ΔCt (N2 – E) between the wild-type (G) and mutation(T) of c.29179.

Differences between the Ct values of N2 and E analyzed by the GeneXpert® System and Cepheid Xpert Xpress SARS-CoV-2 assay kit. Cases negative for N2 (Ct>45) were excluded (n = 34). The Mann–Whitney U test was used for statistical analysis. The median Ct value of the wild-type 29179G was 1.0 (IQR = 1.4), whereas that of mutation 29179T was 9.8 (IQR = 1.6; $p < 0.001$)
